# Route-specific effects of desmopressin on bleeding and hyponatremia after kidney biopsy: meta-analysis of intranasal vs. intravenous administration

**DOI:** 10.3389/fneph.2025.1645418

**Published:** 2025-09-23

**Authors:** Li Zheng, Zhoujun Cai, Lina Shao, Wei Zhang, Bin Zhu, Yan Ren

**Affiliations:** ^1^ Zhoushan Dinghai Central Hospital (Dinghai District of Zhejiang Provincial People’s Hospital), Zhoushan, China; ^2^ Urology & Nephrology Center, Department of Nephrology, Zhejiang Provincial People’s Hospital (Affiliated People’s Hospital), Hangzhou Medical College, Hangzhou, Zhejiang, China; ^3^ Geriatric Medicine Center, Department of Endocrinology, Zhejiang Provincial People’s Hospital (Affiliated People’s Hospital), Hangzhou Medical College, Hangzhou, Zhejiang, China

**Keywords:** desmopressin, kidney biopsy, bleeding events, meta-analysis, hyponatremia

## Abstract

**Background:**

Hemorrhage represents the primary complication associated with kidney biopsy, with post-biopsy bleeding occurring in up to 14% of cases. Some clinicians routinely administer hemostatic agents, such as desmopressin, prior to kidney biopsy to mitigate the risk of significant bleeding. However, the efficacy of this practice remains contentious. Consequently, this meta-analysis was undertaken to assess existing studies regarding the efficacy and safety of desmopressin used before kidney biopsy.

**Methods:**

This systematic review and meta-analysis incorporated both randomized controlled trials and observational studies that examined the outcomes of desmopressin administration prior to percutaneous renal biopsy. Efficacy was measured by the incidence of bleeding events, while safety was assessed through the rate of hyponatremia. A comprehensive search of multiple databases was performed, and the risk of bias was evaluated, and statistical analyses were conducted using appropriate models.

**Results:**

Twelve studies were included. The primary meta-analysis showed no significant reduction in overall bleeding risk with desmopressin (pooled OR 0.71, 95% CI: 0.47 - 1.09; I² = 79%; p = 0.12).Statistically significant differences were observed in the intranasal administration group (pooled OR 0.41;95% CI: 0.28 to 0.60; I ^2^ = 20%; p < 0.0001)(Fixed effect), the RCT group (pooled OR 0.30; 95% CI: 0.17 to 0.53; I ^2^ = 0%; p < 0.0001)(Fixed effect), the low bias group (pooled OR 0.53; 95% CI: 0.32 to 0.87; I ^2^ = 74%; p = 0.01)(Random effect). We conducted statistical analysis on six studies with specific data on hyponatremia, and the pooled OR used fixed model was 2.14 (95% CI: 1.51 to 3.03; I ^2^ = 28%) (Fixed effect), indicating there was a statistical difference between the two groups (p < 0.0001).

**Conclusion:**

Desmopressin did not significantly reduce overall bleeding risk after kidney biopsy. While intranasal administration, RCT only and low bias group showed efficacy in subgroup analyses, it carried a significant hyponatremia risk. Route-specific protocols warrant further study.

**Systematic review registration:**

https://www.crd.york.ac.uk/PROSPERO/, identifier CRD42023391915.

## Introduction

As reported, the incidence of chronic kidney disease (CKD) in China is 8.2% (1).Renal biopsy has been considered the gold standard for the diagnosis of pathologic kidney diseases since the early 1950s (2). This minimally invasive procedure involves extracting a small sample of kidney tissue for microscopic examination. It allows for the precise identification of pathological changes in the glomeruli, which are crucial for determining the type and severity of the disease. The biopsy provides valuable information that guides the selection of appropriate treatment strategies, thereby improving patient outcomes. Despite the potential risks associated with the procedure, its benefits in accurately diagnosing and managing chronic kidney diseases are undeniable.

However, hemorrhage is the main complication of kidney biopsy. Post-biopsy hemorrhage can range from minor hematuria to severe bleeding requiring intervention. Immediate management typically involves monitoring the patient’s vital signs and ensuring they are hemodynamically stable. In some cases, blood transfusions or even surgical intervention may be necessary. As Quan Yao Ho reported, the incidence of gross hematuria is 3.18%, 0.31% require blood transfusion treatment after kidney biopsy, and 1.63% have perirenal hematoma (3). There are significant individual differences in kidney biopsy bleeding. The high-risk group has a bleeding risk 75 times higher than the low-risk group, especially for patients with renal insufficiency (4). The risk of complications after renal biopsy in hospitalized patients is higher than previously reported, and several factors such as low platelet count, female gender, and high BUN are associated with this risk (5). Therefore, efforts are being made to reduce the incidence of kidney biopsy bleeding.

Desmopressin, also known as deamino-8-D-arginine vasopressin (DDAVP), is a synthetic analog of the hormone vasopressin. It is used to treat conditions like diabetes insipidus and hemophilia by reducing urine output and promoting blood clotting, respectively. Administered as a nasal spray or injection, desmopressin helps manage fluid balance and supports the body’s natural healing processes. desmopressin is utilized in the context of renal biopsy to mitigate bleeding risks, particularly in patients with compromised platelet function or those with uremia. It functions by elevating levels of von Willebrand factor and factor VIII, which aid in clotting and reduce bleeding time (6, 7). Some doctors routinely use hemostatic drugs such as desmopressin to prevent major bleeding before kidney biopsy, but whether they are effective is debatable. There is insufficient evidence to support the benefit or harm of desmopressin use pre-renal biopsy to reduce the risk of post-renal biopsy bleeding (8).

Earlier systematic reviews (9)(e.g., Lim et al., 2021) were inconclusive due to critical limitations: (1) inclusion of only 2 RCTs (n=197) with underpowered analyses; (2) failure to stratify by administration routes, despite known pharmacokinetic differences between intranasal/IV formulations; (3) exclusion of observational studies, thereby ignoring real-world safety signals like hyponatremia.

In recent years, there have been related research reports, therefore, this Meta-analysis focuses on the existing studies to see whether using desmopressin before kidney puncture can reduce bleeding or not, and to study the safety of hemostatic drugs.

## Methods

This systematic review and meta-analysis was registered on PROSPERO (CRD4202339 1915) and is reported according to the Preferred Reporting Items for Systematic Reviews and Meta-Analyses guidelines (10).

### Study inclusion criteria

#### Types of studies and study participants

##### Inclusion criteria

1. patients who underwent a kidney biopsy with desmopressin before the procedure.
2. randomized controlled trials, cohort studies, or case-control studies, Retrospective study.
3. the article published in English or Chinese.

##### Exclusion criteria

Abstract only (no accompanying paper);no complication data were provided; no control group was included; biopsy for kidney mass; open kidney biopsy; nonkidney biopsy; review or editorial; patient report; patients on dialysis; and use of a transjugular approach.

#### Types of outcome measures

Bleeding complications comprised the primary outcomes. Major bleeding complications were defined as those resulting in permanent adverse sequelae or death or those requiring interventional therapy—medical (including blood transfusions), radiological, or surgical (such as embolization or coiling). Minor complications were defined as hematuria or hematomas requiring no active intervention. Adverse events related to DDAVP administration were those such as headache, facial flushing, hypotension, tachycardia and hyponatremia, or any others identified by included studies.

### Data source

We searched captured articles published on PubMed, Embase, the Cochrane Library, and the WanFangData up to October 28, 2024 without language, publication year or publication status restrictions. 7734 records were found in the database and 9 trials were registered on the International Clinical Trials Registry Platform. We attempted to contact the research leader via email to inquire about research trends, but one person replied that the article had not been published yet and could not provide raw data, while the others did not reply.

### Study selection

**Figure 1:**
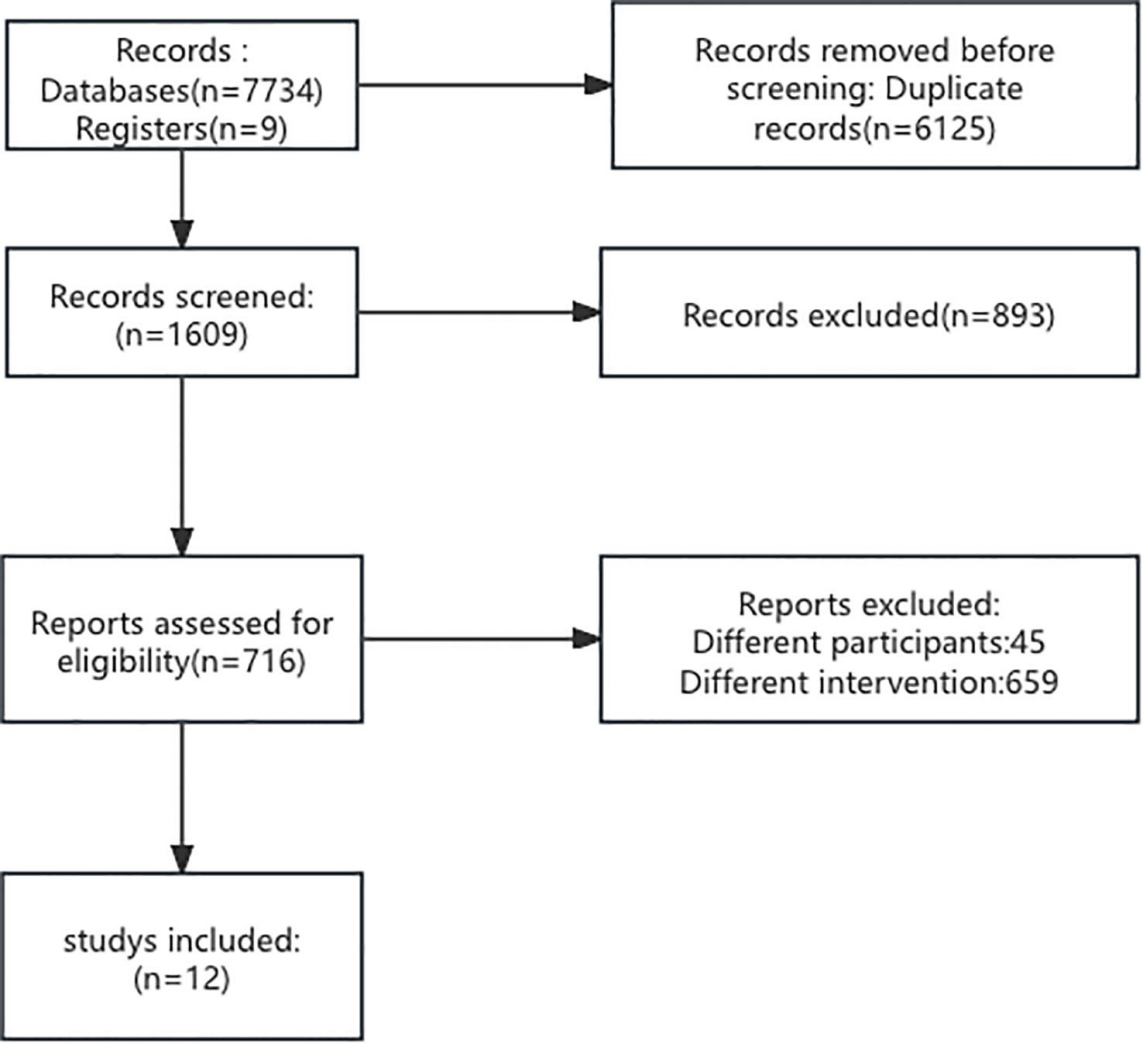
Flowchart depicting the selection process of studies. Out of 7,743 records from databases and registers, 6,125 duplicates were removed. From 1,609 screened records, 893 were excluded. 716 reports were assessed for eligibility, with 704 excluded for different participants or interventions, resulting in 12 studies included.

The papers were randomly divided among 2 reviewers. The reviewers (LZ and ZJC) chose the articles according to the inclusion criteria. In the full-text review, papers were again randomly divided and evaluated by three reviewers. The entire paper was assessed, and the reasons for exclusion were recorded in detail for each paper. For full data abstraction, the papers were randomly assigned to two reviewers. The disagreements were sent back as queries to the original two reviewers who then discussed and resolved them via consensus. Finally, 12 papers were included in this meta-analysis. The data were recorded into Excel (**Table 1**). The flowchart is shown in **Figure 1**.

**Table 1:** Characteristics of included studies (N=12).

	First author, Year, Ref	setting	Study type	N	male/ female	age	Participants	Intervention	Intervention (control group)	Primary outcomes	Risk of bias
1	Sattari SA 2022 (11)	Two centres, Iran	RCT	120patients [60=intervention; 60= control]	31/27	45.29 ± 15.95	reduced kidney function 15 < eGFR < 90 mL/min/1.73 m^2^	3 ug/kg (intranasal)	1mL/kg sodium chloride 0.65% (intranasal)	bleeding complications	low
2	Ho QY 2020 (12)	Single centre, Singapore	retrospective cohort study	195patients [98=intervention; 97= control]	51/58	50.6 (15.3) / 50.6 (13.8)	renal allograft biopsies in adult (≥21 years old) (serum creatinine ≥150 µmol/L)	0.3 μg/kg (administered)	NA	bleeding	low
3	Lim CC 2019 (13)	a single-center Singapore	retrospective cohort study	436patients [226=intervention; 210= control]	120/128	51.3 (42.3, 60.0)	serum creatinine≥ 150 µmol/L	median dose 0.20 (0.17, 0.24) µg/kg intravenous	NA	severe hyponatremia (serum sodium≤125 mmol/L) within 7 days post-biopsy. Secondary outcome was post-biopsy bleeding	moderate
4	Peters B 2015 (14)	multicenter , Sweden	prospective	576patients [204=intervention; 372= control]	151/256	median age 61 years	creatinine above 150 umol/L	0.3ug/kg subcutaneous	NA	Biopsy complications	low
5	Leclerc S 2020 (15)	a single-center Canada	retrospective cohort study	413patients [328=intervention; 85= control]	184/42	median age61 years (IQR, 64–70 years)	all patients, regardless of eGFR and other comorbidities.	0.3 ug/kg iv	NA	bleeding complications	moderate
6	Athavale A 2019 (16)	a single-center America	retrospective cohort study	269patients [100=intervention; 169= control]	45/80	median age46.05 VS48.22	All consecutive patients	0.3ug/kg intravenous 30 min prior to biopsy	NA	composite of hemoglobin drop ≥1 g/dL, hematoma on post biopsy ultrasound, gross hematuria, erythrocyte transfusion or angiography to stop bleeding	moderate
7	Jose L 2021 (17)	a single-center India	a retrospective study	432patients [307=intervention; 125= control]	193/82	39.1 ± 15.3	[eGFR] <30 mL/min/1.73 m^2^	dose of intranasal desmopressin used was 40 µg puffs in each nostril, given twice – the first dose an hour before and the second one 15 minutes before the biopsy (cumulative dose of desmopressin = 160 µg).	NA	bleeding complication	low
8	Cheong M 2022 (18)	single-center study; South Korea	retrospective study	1254patients [627=intervention; 627= control]	337/330	49.0 ± 16.7 VS 49.2 ± 16.0	aged 18 years or above. excluding for transplant kidney biopsy ,mass biopsy and open kidney biopsy	a dose of 0.3ug/kg desmopressin in 100 cc normal saline 30min before the procedure.	NA	Bleeding events	low
9	Rao NS 2020 (19)	single-center, interventional study India	prospective and retrospective	194patients [89 = intervention; 105= control]	not mentioned	36.0 ± 16.8 VS 40.7 ± 16.8	serum creatinine >132.6 umol/L and/or eGFR was <60 mL/min/1.73 m^2^	intranasal desmopressin was administered at a dose of 150 ug 1 h prior to the planned renal biopsy	NA	Bleeding complications	low
10	Manno C 2011 (20)	Single centre, Italy	RCT	162 patients [80 = intervention; 82 = control]	45/43	40.6±14.6	native kidney	subcutaneously, dosage of 0.3 ug/kg 1 hour before the kidney biopsy	subcutaneously 1 mL of saline solution	incidence of postbiopsy bleeding complications	low
11	Carroll M 2023 (21)	Single centre, Australia	retrospective medical record review	153 patients [54 = intervention; 99 = control]	not mentioned	not mentioned	native renal biopsies	not mentioned	not mentioned	bleeding complications	moderate
12	Barrios RHS 2021 (22)	single-centre study, Spain	a retrospective study	130patients [65 = intervention; 65 = control]	43/42	54.5 (IQR 47–66) VS 54 (IQR 45–66.5)	all renal biopsies	administered intravenously as a single dose of 0.3 ug/kg within 1 hour of the procedure.	NA	Safety of renal biopsy bleeding prophylaxis with desmopressin	moderate

### Quality assessment and risk of bias assessment

The quality of the literature was evaluated by the RoB 2 (Revised Cochrane risk-of-bias tool for randomized trials) (23). The RoB 2 tool encompasses five domains: ‘randomization process, deviations from intended interventions, missing outcome data, measurement of the outcome, and selection of the reported result’. Based on the responses, each study was categorized as having low, some concerns, or high risk of bias. The Newcastle Ottawa (NO) scale was used for assessing observational studies (24). The NO scale encompasses selection, comparability, and outcome domains. Based on the responses, each study was categorized as having good/poor quality.

### Data analysis

For each study, we collected descriptive data, which included the sample size as well as participants’ ages and sex. The authors also collected clinical data, including whether the study design was a randomized controlled trial(RCT), types of treatment and control.

For dichotomous outcomes, we would calculate the OR and relative 95% CI. Missing dichotomous outcome data would be handled according to the intention-to-treat principle. Participants who dropped out after randomization will be considered having a negative outcome. I^2^ > 75% is considered substantial heterogeneity. The fixed effects model was used if the P value of the Q test was ≥ 0.1 or I^2^< 50%. Otherwise, the random effects model was applied to pool the outcomes. We performed a meta-analysis using Revman5.3.

## Results

### Characteristics of the included studies

This study ultimately enrolled 12 studies, including 2 randomized controlled trials, 8 retrospective cohort studies, and 2 prospective studies. Sample sizes ranged from 120 to 1254. Two studies did not mention the gender ratio, and the remaining 10 studies included 1200 males and 1088 females, with males being the majority. Most studies were single-center studies(10 out of 12). Most studies (7 out of 12) had a low risk of bias, while 5 studies had a moderate risk. There were 12 studies from four continents, including 6 in Asia (1 in Iran, 2 in Singapore, 2 in India, and 1 in South Korea), 2 in North America (1 in Canada and 1 in the United States), 3 in Europe (1 in Sweden, 1 in Italy, and 1 in Spain), and 1 in Oceania (1 in Australia) (shown in **Table 1**) (11–22).

### Efficacy parameters

#### Number of bleeding events

**Figure 2:**
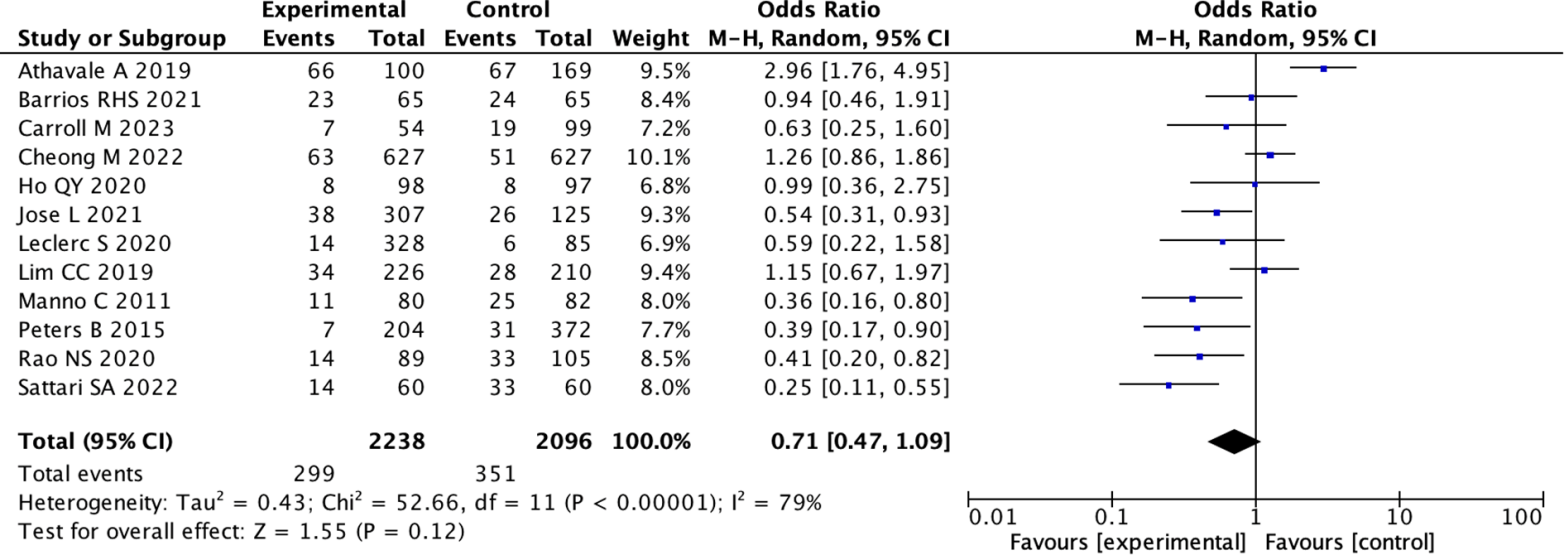
Forest Plot of Odds Ratio in number of bleeding events with desmopressin vs control. Most studies show varying odds ratios, with Athavale A 2019 having the highest odds ratio of 2.96, favoring the experimental group. The overall effect shows an odds ratio of 0.71, suggesting a slight favor towards the control. Heterogeneity is high with I-squared at 79 percent.

**Figure 3:**
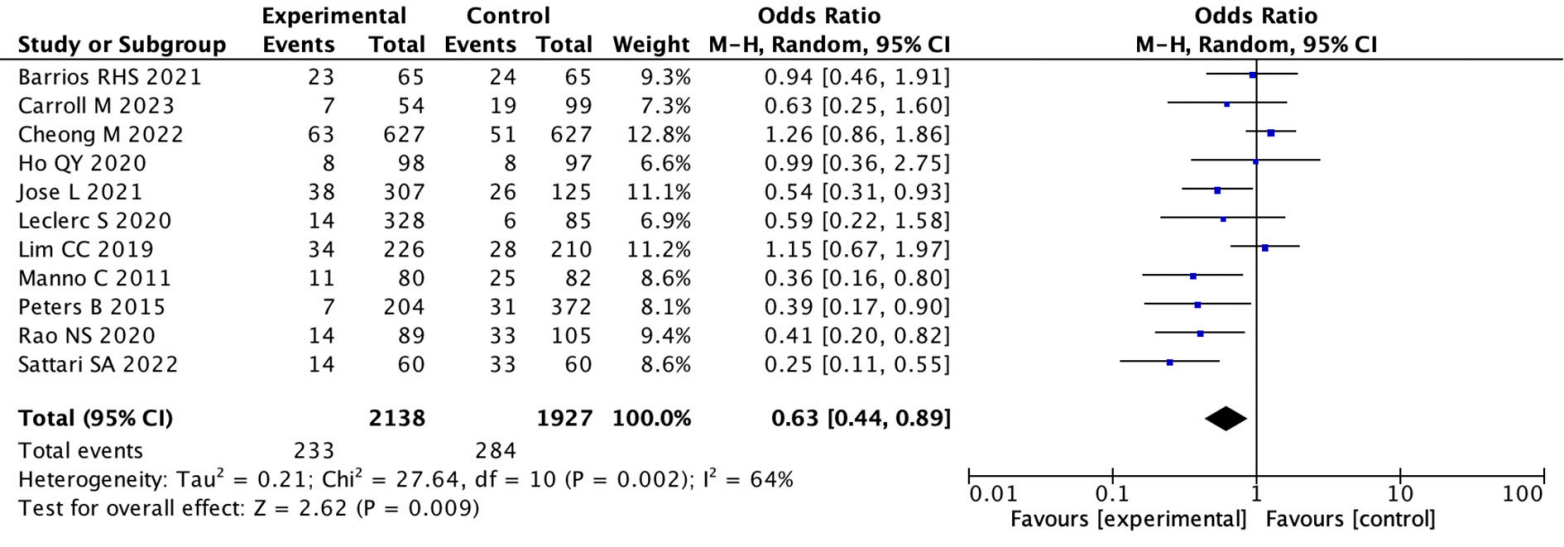
Forest plot showing a meta-analysis of 11 studies comparing experimental and control groups. It lists sample sizes, events, weights, and odds ratios with confidence intervals. The combined odds ratio is 0.63, with significant heterogeneity. A diamond at the bottom represents the overall effect, favoring the experimental group.

**Figure 4:**
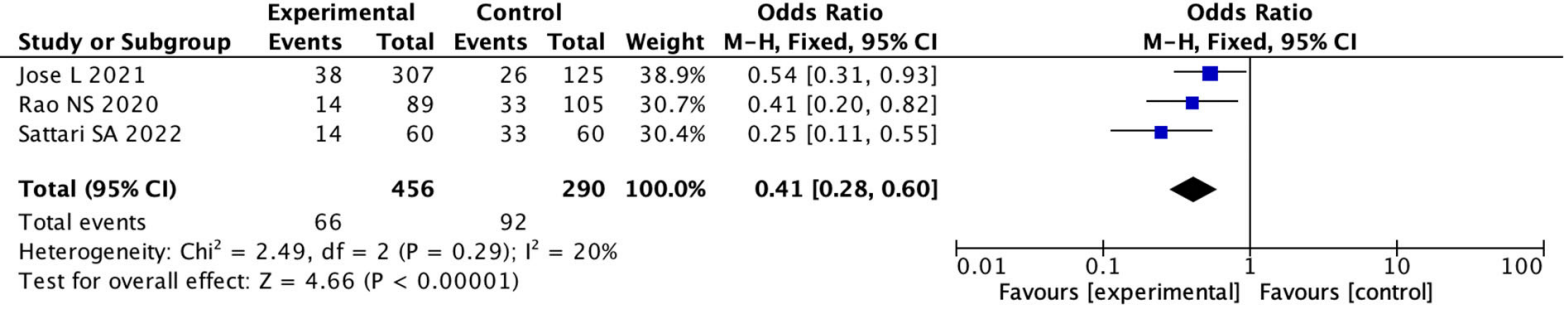
Forest Plot of Odds Ratio in number of bleeding events with desmopressin vs control intranasal Odds ratios with 95% confidence intervals for each study are shown, summarized as a diamond. Studies include Jose L 2021, Rao NS 2020, and Sattari SA 2022, with odds ratios of 0.54, 0.41, and 0.25, respectively. The overall odds ratio is 0.41, indicating a significant favor towards the experimental group. Heterogeneity is low with a Chi-squared of 2.49, degrees of freedom of 2, and an Isquared of 20%. The Z-test for overall effect shows significance with a p-value less than 0.00001.

**Figure 5:**
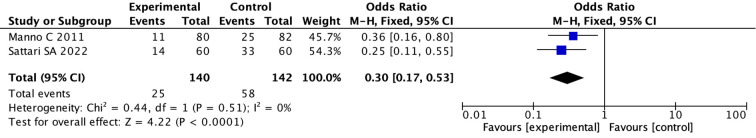
Forest plot showing meta-analysis results from two RCT studies: Manno C 2011 and Sattari SA 2022. The odds ratios are 0.36 and 0.25, respectively, favoring experimental over control. The total odds ratio is 0.30 with a 95% confidence interval of 0.17 to 0.53. Heterogeneity is low with Chi-square equals 0.44 and I-squared equals 0 percent. Overall effect is significant with Z equals 4.22 and P less than 0.0001.

**Figure 6:**
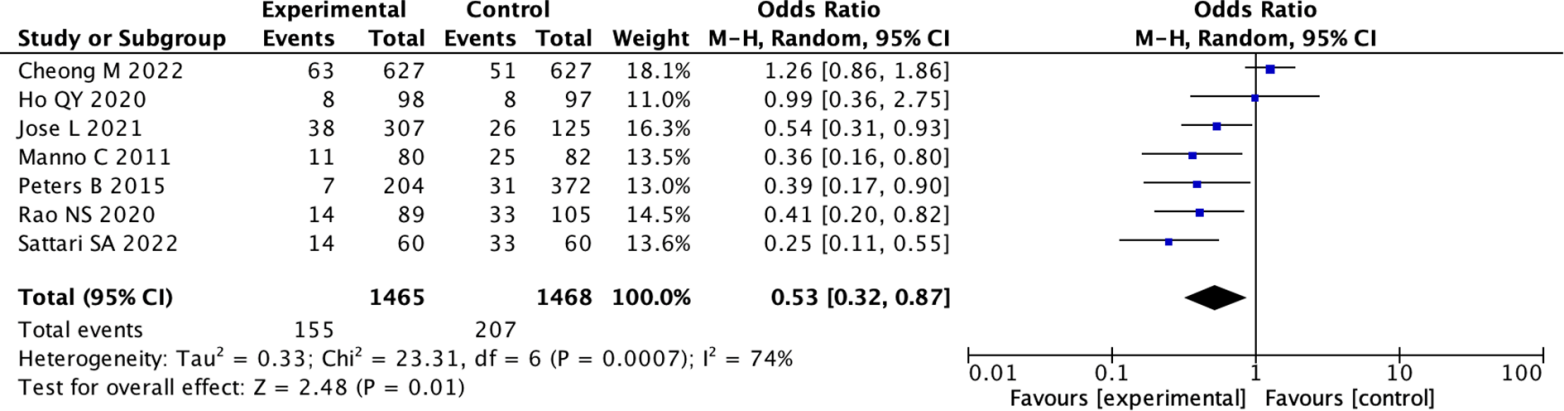
Forest Plot of Odds Ratio in number of bleeding events with desmopressin vs control low bias. Studies favoring the experimental group have odds ratios below one. The overall effect shows a combined odds ratio of 0.53, favoring the experimental group, with significant heterogeneity (I squared equals seventy-four percent, P equals zero point zero one).

(1) All 12 studies mentioned the situation of bleeding events after kidney biopsy. Out of 2238 cases using desmopressin, there were 299 bleeding events, while in the control group of 2096 cases, there were 351 cases of kidney biopsy bleeding. Due to the high heterogeneity, we adopted a random effects model. However, the primary meta-analysis showed no significant reduction in overall bleeding risk with desmopressin (pooled OR 0.71, 95% CI: 0.47 – 1.09; I² = 79%; p = 0.12) (**Figure 2**) (**Supplementary Figure 1**). Therefore, we conducted subgroup analysis again.

(2) We assessed robustness through sensitivity analysis excluding individual studies.After excluding one outlier study (Athavale et al., 2019) we found that the pooled OR was 0.63 (95% CI: 0.44 to 0.89; I ^2^ = 64%)(random effects model) in the remaining 11 studies. This indicates that the desmopressin group had a significant decrease in bleeding events compared to the control group (p = 0.009) (**Figure 3**) (**Supplementary Figure 2**).

(3) According to the renal function of the enrolled patients, they were divided into the general population and the population with renal insufficiency. Among the enrolled patients, there were 3 studies involving the general population, 8 studies focusing on patients with renal dysfunction, and 1 study with unknown renal function status. In the general population, the pooled OR was 0.89 (95% CI: 0.21 to 3.73; I ^2^ = 91%)(random effects model), indicating that there was no difference between the two groups (p = 0.87)(**Supplementary Figures 3**, **4**). While in the population with renal insufficiency, the pooled OR (using the random effects model) was 0.66 (95% CI: 0.43 to 1.02 ;I ^2^ = 71%), indicating that there was no statistical difference between the two groups. (p = 0.06) (**Supplementary Figures 5**, **6**).

(4) We conducted subgroup analysis of 16g and 18g based on the size of the kidney biopsy needle. Three studies used 18g kidney biopsy needles, while the majority of five studies used 16g needles. In the 18g group, the pooled OR was 0.75 (95% CI: 0.18 to 3.15; I ^2^ = 94%)(random effects model), indicating there was no difference between the two groups (p = 0.69) (**Supplementary Figures 7**, **8**). While in the group using 16g needles, the pooled OR (using the random effects model) was 0.64 (95%CI: 0.38 to 1.07; I ^2^ = 54%), indicating there was no statistical difference between the two groups (p = 0.09) (**Supplementary Figures 9**, **10**).

(5) We divided the studies into nasal spray subgroup and intravenous medication subgroup based on medication method. Three studies used nasal spray administration, and seven studies used intravenous administration. In the intravenous group, the pooled OR was 0.88 (95%CI: 0.49to 1.57; I ^2^ = 80%)(random effects model), indicating there was no difference between the two groups (p = 0.67) (**Supplementary Figures 11**, **12**). While in the population using nasal spray administration, the pooled OR used fixed model was 0.41 (95% CI: 0.28 to 0.60; I ^2^ = 20%), indicating there was a statistical difference between the two groups (p < 0.0001) (**Figure 4**; **Supplementary Figure 13**).

(6) We divided the studies into RCT group and non-RCT group according to the research design. Two studies were RCT, the pooled OR was 0.30 (95%CI: 0.17 to 0.53; I ^2^ = 0%)(fixed effects model), indicating there was a statistical difference between the two groups (p < 0.0001) (**Figure 5**; **Supplementary Figure 14**). However, in non-RCT studies, the pooled OR used random model was 0.85 (95% CI: 0.56 to 1.29; I ^2^ = 75%)(random effects model), indicating there was no difference between the two groups (p = 0.45) (**Supplementary Figures 15**, **16**).

(7) We divided the studies into low-risk group and medium-risk group according to the risk of bias score. Five studies were of moderate risk, the pooled OR was 1.11 (95% CI: 0.60 to 2.06; I ^2^ = 74%) (random effects model) (**Supplementary Figures 17**, **18**), indicating there was no difference between the two groups (p = 0.74). While in low risk studies, the pooled OR used random model was 0.53 (95% CI: 0.32 to 0.87; I ^2^ = 74%), indicating there was a statistical difference between the two groups (p = 0.01) (**Figure 6**; **Supplementary Figure 19**).

#### Safety parameters

**Figure 7:**
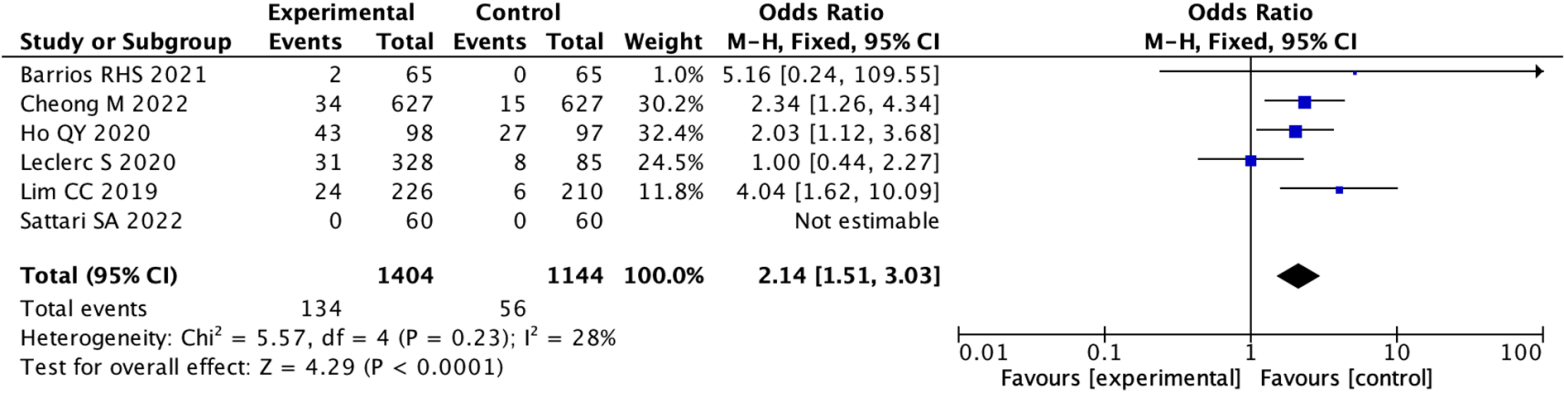
Forest Plot of Odds Ratio in number of hyponatremia events with desmopressin vs control. The overall odds ratio is 2.14 with a confidence interval of 1.51 to 3.03, indicating a significant effect favoring the experimental group. Individual study odds ratios vary, with Cheong M 2022 and Ho QY 2020 showing significant results. Heterogeneity is low (I. = 28%). The pooled effect size is represented by a diamond.

Specific data on the incidence of hyponatremia were found in 6 studies, while another 6 studies mentioned the presence of a transient (1 hour) mild increase (5%) in heart rate. In addition, three patients reported transient mild headaches, two patients developed self-resolving cutaneous flushing, and one case developed transient self-resolving tachycardia in another study. We performed statistical analysis on six studies that provided specific data on hyponatremia, the pooled OR used fixed model was 2.14 (95% CI: 1.51 to 3.03; I ^2^ = 28%), indicating there was a statistical difference between the two groups. (p < 0.0001) (**Figure 7**; **Supplementary Figure 20**).

## Discussion

Kidney biopsy is considered to be the gold standard for diagnosing chronic kidney disease (CKD) and is crucial for developing treatment strategies (25). However, this invasive procedure carries the risk of bleeding complications (3). The use of desmopressin prior to the biopsy has been proposed to mitigate this risk, yet the evidence remains inconclusive. In this meta-analysis, we aim to synthesize the existing research findings to evaluate the efficacy of desmopressin in reducing bleeding complications associated with kidney biopsies. By analyzing data from multiple studies, we will assess the overall impact of desmopressin on bleeding risk and its implications for clinical practice. Our results may provide valuable insights for nephrologists regarding the pre-biopsy management of patients, potentially boosting the safety of this essential diagnostic procedure.

However, after analyzing 12 studies, we found something. While there appears to be a trend toward reduced bleeding rates with the use of desmopressin, this difference did not reach statistical significance. Therefore, we cannot conclude that desmopressin effectively decreases bleeding complications associated with kidney biopsies. The lack of a conclusive finding may be attributed to the considerable heterogeneity among the included studies (I ^2^ = 79%), which could influence the reliability of our results. The substantial heterogeneity likely stemmed from: 1. Clinical diversity: Varied definitions of bleeding events (e.g., minor hematuria vs. transfusion-requiring hemorrhage), needle gauges (16G vs. 18G), and patient risk profiles. 2. Methodological biases: 10 of 12 included studies were observational, risking confounding by indication (e.g., desmopressin preferentially given to high-bleeding-risk patients). 3. Route-dependent pharmacokinetics: Differing bioavailabilities of intranasal (10 – 20%) vs. IV (100%) formulations may partly explain subgroup contrasts, but dose-response relationships could not be examined. Therefore, we conducted a very detailed subgroup analysis.

We assessed robustness through sensitivity analysis excluding individual studies. In our analysis of the 12 studies, we identified the study by Ambarish Athavale in 2019 exhibited substantial bias, with a relatively high moderate risk of bias score. After excluding one outlier study (Athavale et al., 2019), a *post-hoc* analysis suggested a potential signal toward reduced bleeding (pooled OR 0.63, 95%CI: 0.44 – 0.89), though this remains secondary to the primary null finding and requires validation in dedicated trials. Additionally, the heterogeneity among the remaining studies decreased significantly, indicating a more consistent effect across these analyses (pooledOR 0.63; 95%CI: 0.44 to 0.89; I ^2^ = 64%; p=0.009). The null findings regarding desmopressin’s efficacy should be interpreted with caution. Our in-depth analysis identified critical methodological concerns: First, confounding by indication likely occurred through preferential DDAVP administration to high-bleeding-risk patients, potentially obscuring therapeutic benefits. Second, unaddressed population heterogeneity was evident—while renal-impaired patients (serum creatinine ≥1.8 mg/dL) might benefit, low-risk cohorts exhibited paradoxically increased bleeding. Third, the composite endpoint’s susceptibility to hemodilution effects (particularly hemoglobin drop ≥1 g/dL) may have inflated bleeding rates artifactually. These limitations collectively explain the counterintuitive conclusion.

This finding supports the potential benefit of desmopressin in minimizing bleeding risks during kidney biopsies and suggests that it may be a valuable consideration in the pre-procedural management of patients.

It is well-known that renal insufficiency is a significant risk factor for bleeding complications during kidney biopsy procedures. Professor Ragnar Palsson, in his study (26), also noted that kidney biopsy is generally well-tolerated by most individuals. However, he identified that an estimated glomerular filtration rate (eGFR) of less than 30 mL/min is a risk factor for postoperative hemoglobin decline. This highlights the importance of assessing renal function prior to the procedure, as patients with severely compromised kidney function may be at an increased risk for complications. Therefore, we divided participants in the study into subgroups of individuals with renal insufficiency and the general population for subgroup analysis. However, no statistical differences were found. This is inconsistent with our expectations. It may be because patients with renal insufficiency have limited response to drugs, and the protective effect of the drug is less than the risk of bleeding caused by renal insufficiency, so the outcome was not altered.

As concluded in Tingting Zhan’s study (27), the use of a 16G puncture needle is associated with a higher overall incidence of complications compared to an 18G needle. Consequently, we classified the studies based on the type of puncture needle used. Because I^2^ is more than 50, using the Random model, it can be seen that the use of desmopressin before kidney biopsy seems to be beneficial in preventing postoperative bleeding, but the statistical difference has not been reached (**Supplementary Figure 9**). Further expansion of the sample size is needed to determine whether desmopressin has a protective effect before kidney biopsy.

The use of desmopressin in the field of nephrology has a long history. Common administration methods include intravenous injection, nasal spray, and oral administration. Due to the differences in administration routes and dosages, and to reduce heterogeneity among studies, we categorized the studies into two groups: intravenous administration (0.3 µg/kg) and nasal spray. As a result, we found the effect of desmopressin was more pronounced in the nasal spray medication group (**Figure 4**). Nasal sprays are more effective because they avoid the first-pass effect.

Because of the more rigorous design of randomized controlled trials (RCTs), the results are more reliable. We found that the conclusions in RCT studies were consistent, indicating that desmopressin can reduce bleeding complications associated with kidney biopsies. Similar conclusions were also observed in the low-bias group (**Figures 5**, **6**).

desmopressin is an antidiuretic drug that augments the kidney’s reabsorption of water, particularly in the collecting ducts. The following are some of the main reasons for low sodium levels (hyponatremia) associated with desmopressin. desmopressin increases the reabsorption of water in the renal tubules, leading to water retention in the body. This can give rise to plasma dilution, which in turn causes hyponatremia. When desmopressin is used, if the patient consumes excessive amounts of water, the occurrence of hyponatremia may be exacerbated. As the kidneys cannot effectively excrete the excess water, the sodium concentration in the blood decreases. The risk factors for hyponatremia include advanced age, presence of comorbidities, use of concurrent medications, and a reduction in estimated glomerular filtration rate (eGFR) (28).

The primary strength of our review lies in its rigorous methodological approach, thorough search strategies, compliance with PRISMA guidelines, and detailed assessment of the risk of bias. Nonetheless, several limitations should be recognized. We identified a potential risk of publication bias, as indicated by the asymmetrical funnel plot and subgroup analysis. Furthermore, the high heterogeneity observed in some of our analyses, even when employing a random-effects model, suggests significant variability among the included studies that cannot be attributed solely to random chance.

Therefore, we are still unable to conclude that desmopressin can effectively improve kidney biopsy bleeding. Randomized controlled trials comparing desmopressin to placebo are necessary to guide clinical decision-making. These trials should encompass patients with different levels of acute and chronic kidney impairment and should address both native and transplant biopsy contexts. Renal centers that are currently administering desmopressin prior to renal biopsies should undertake a prospective observational study to evaluate all patient outcomes following desmopressin administration. Key outcomes to assess include bleeding complications, the size of hematomas, length of hospital stay, and any complications related to desmopressin treatment.

## Conclusion

This meta-analysis found no statistically significant reduction in overall bleeding risk with desmopressin after kidney biopsy (pooled OR 0.71, 95%CI: 0.47 – 1.09; p=0.12). Although subgroup analyses suggested potential benefits in specific contexts—such as intranasal administration (OR 0.41, 95%CI: 0.28 – 0.60) and RCT settings (OR 0.30, 95%CI: 0.17 – 0.53)—these findings require cautious interpretation due to heterogeneity and limited generalizability. Furthermore, desmopressin use was associated with a significant increase in hyponatremia risk (OR 2.14, 95%CI: 1.51 – 3.03). Given the uncertain efficacy in the broader population and clear safety concerns, routine pre-biopsy desmopressin cannot be recommended. Intranasal desmopressin should be prioritized in future trials targeting high-bleeding-risk kidney biopsy patients. Route-specific protocols should be explored in rigorously designed trials targeting high-risk subgroups.

## Data Availability

The original contributions presented in the study are included in the article/**Supplementary Material**. Further inquiries can be directed to the corresponding author.
