# Supplementary figures and images for: Route-specific effects of desmopressin on bleeding and hyponatremia after kidney biopsy: meta-analysis of intranasal vs. intravenous administration
Source: Front Nephrol. 2025 Sep 23;5:1645418. doi: 10.3389/fneph.2025.1645418 (PMC12500430; doi:10.3389/fneph.2025.1645418)

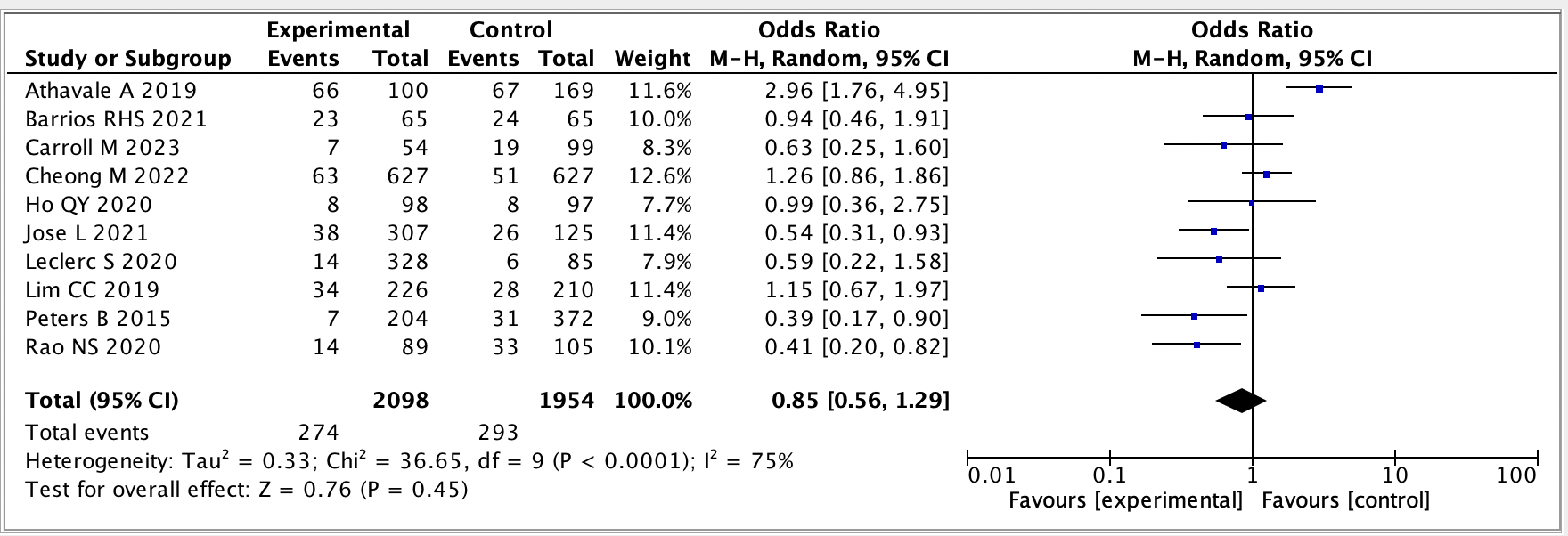

Supplement: Supplementary file 1 [file Presentation1.zip › Image 15.png]

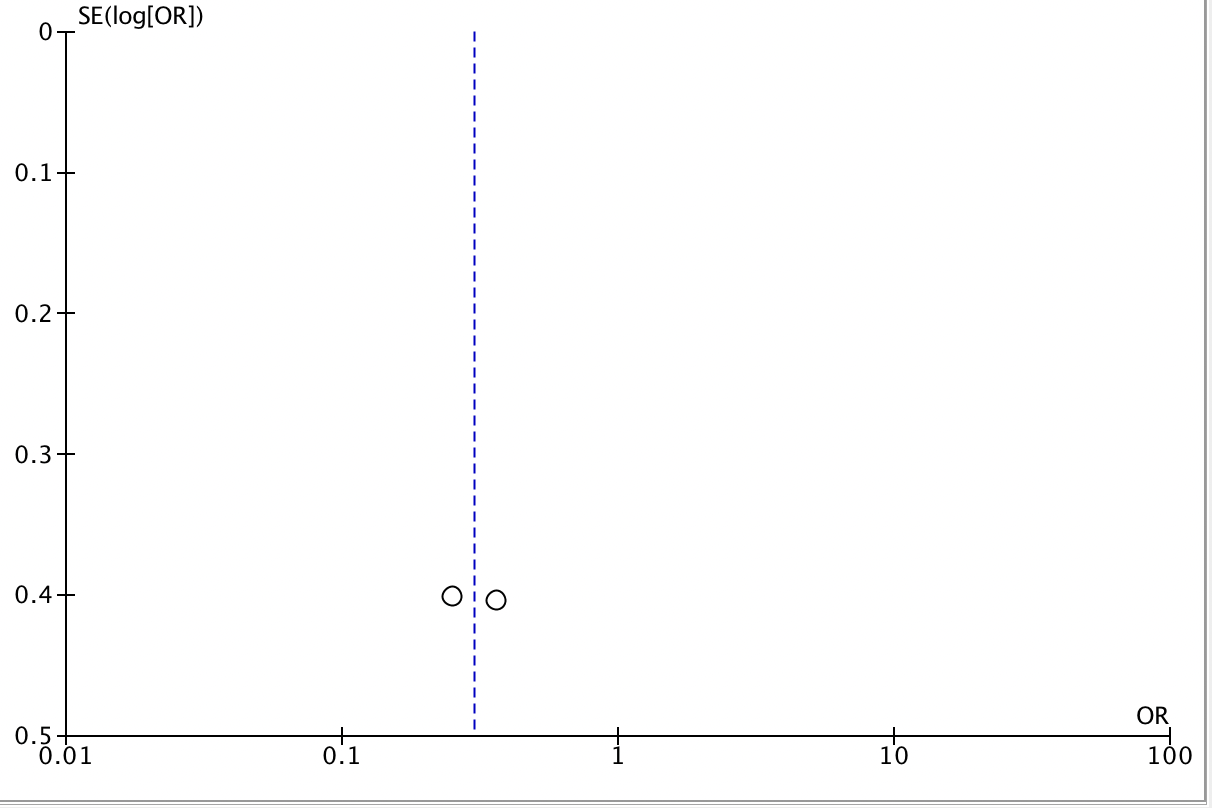

Supplement: Supplementary file 1 [file Presentation1.zip › Image 14.PNG]

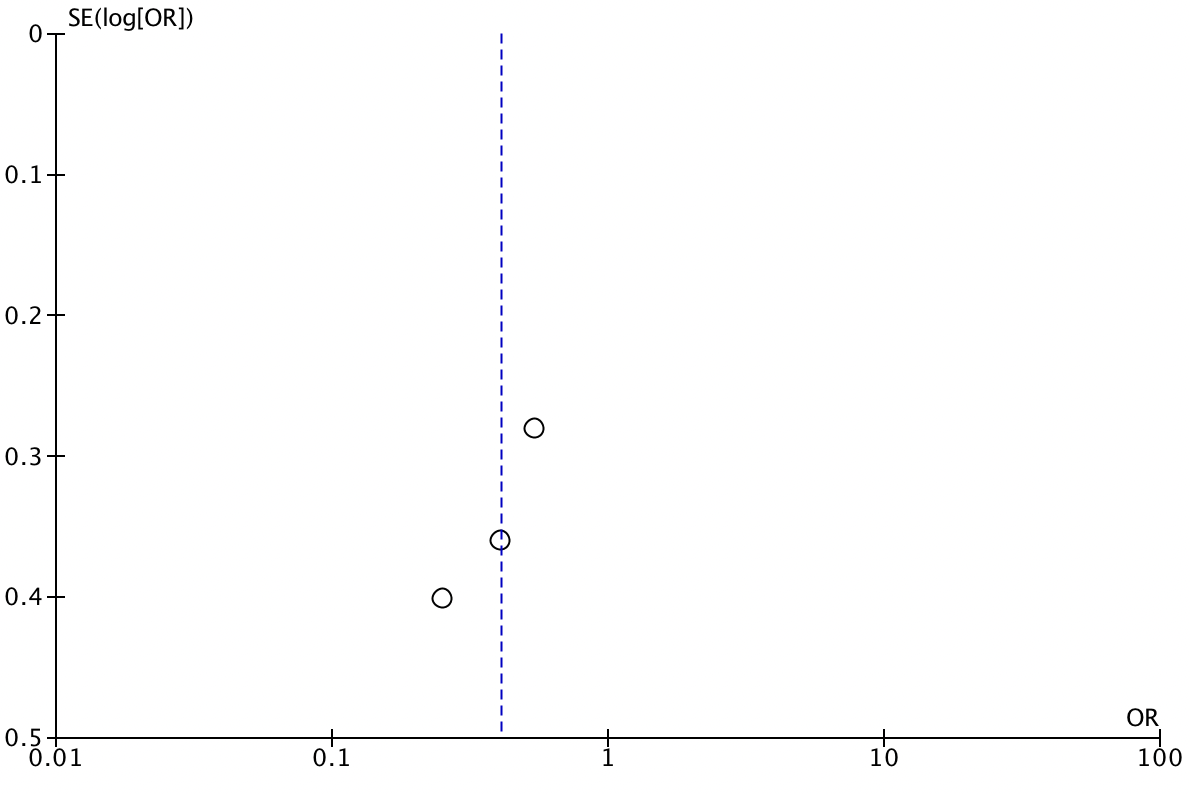

Supplement: Supplementary file 1 [file Presentation1.zip › Image 13.PNG]

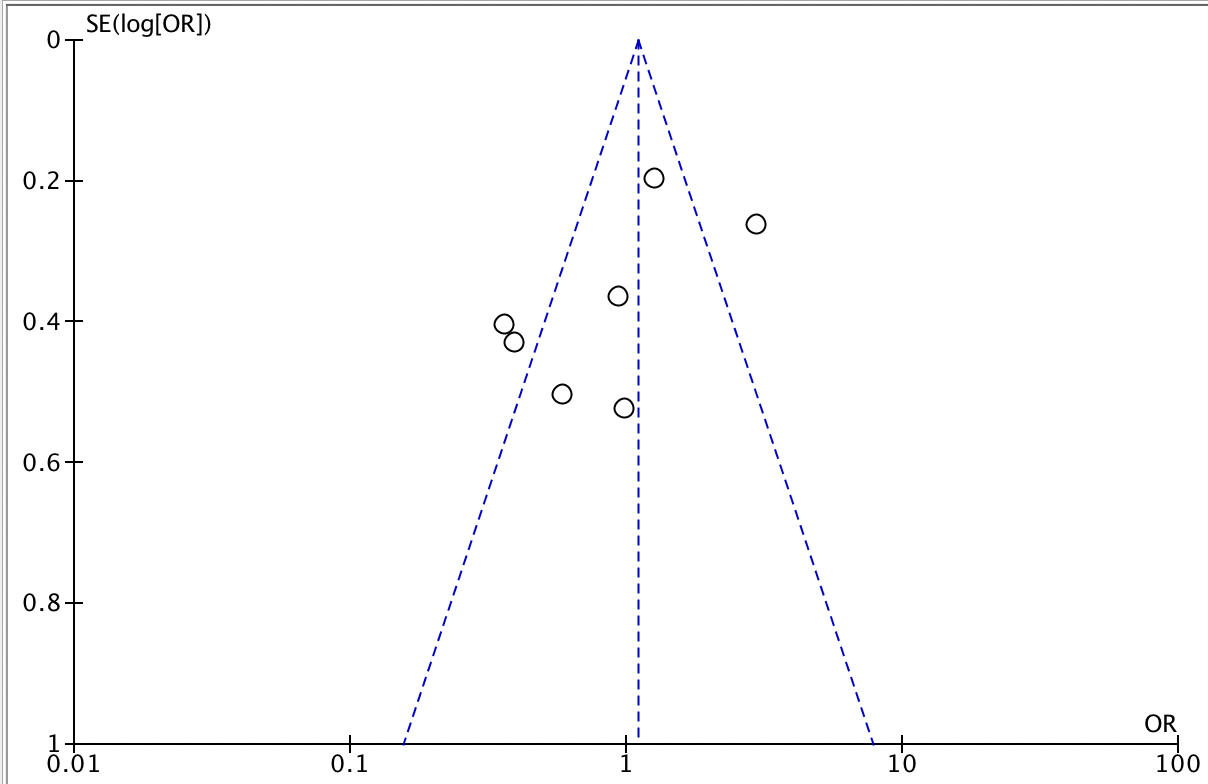

Supplement: Supplementary file 1 [file Presentation1.zip › Image 12.PNG]

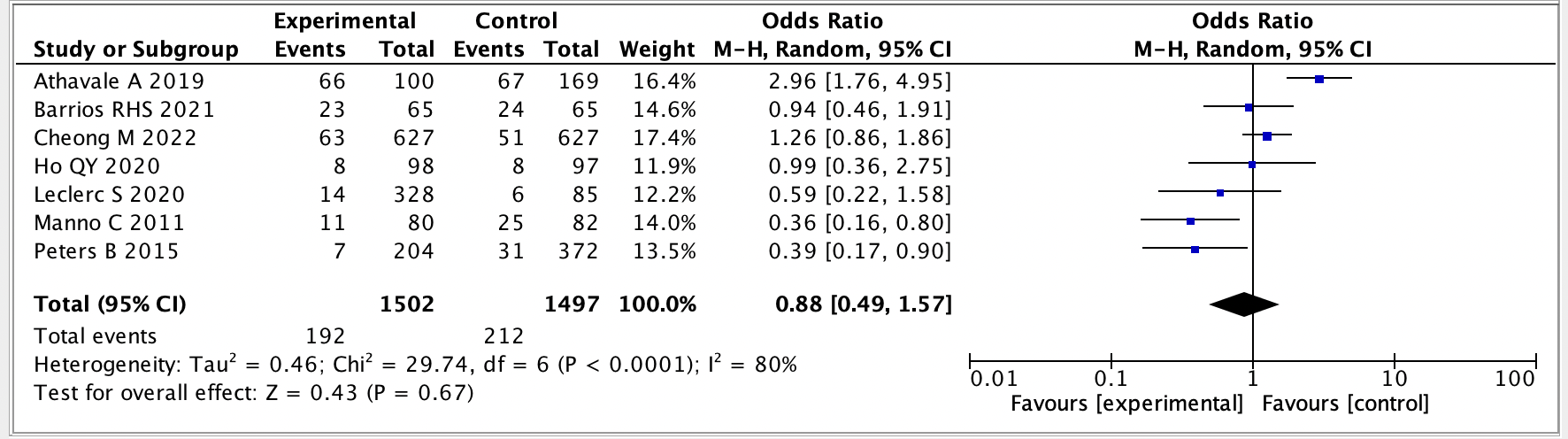

Supplement: Supplementary file 1 [file Presentation1.zip › Image 11.PNG]

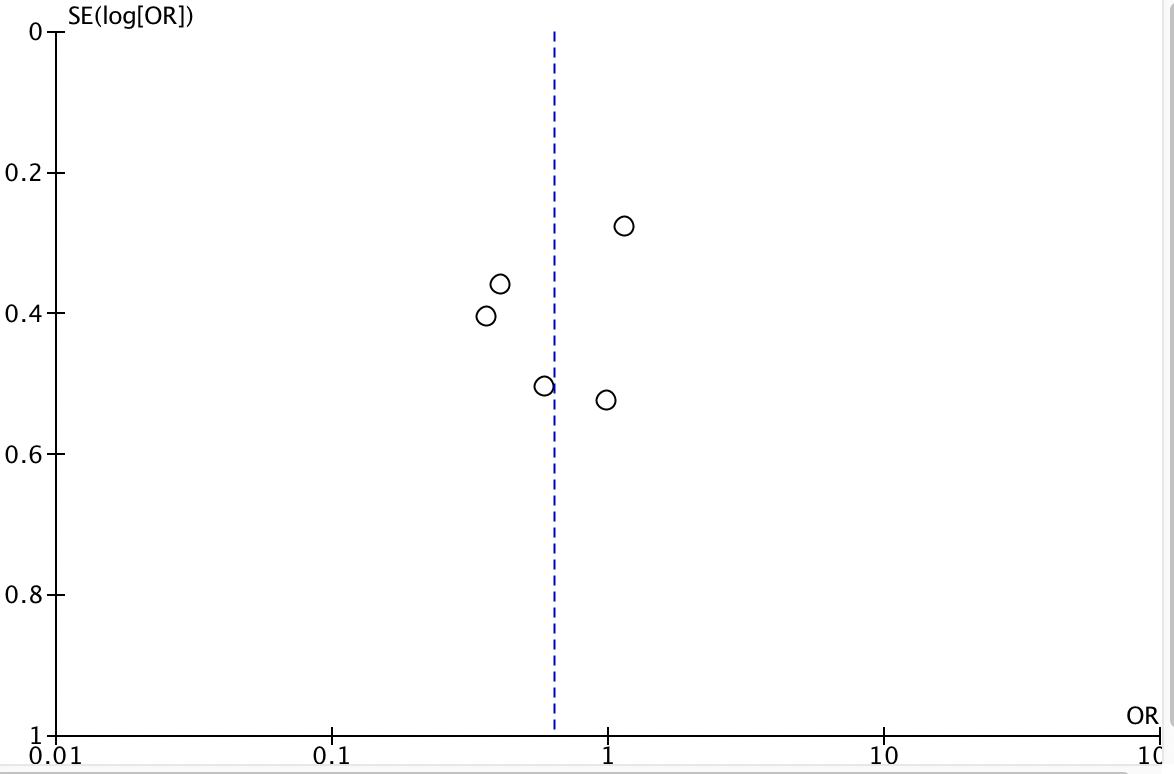

Supplement: Supplementary file 1 [file Presentation1.zip › Image 10.PNG]

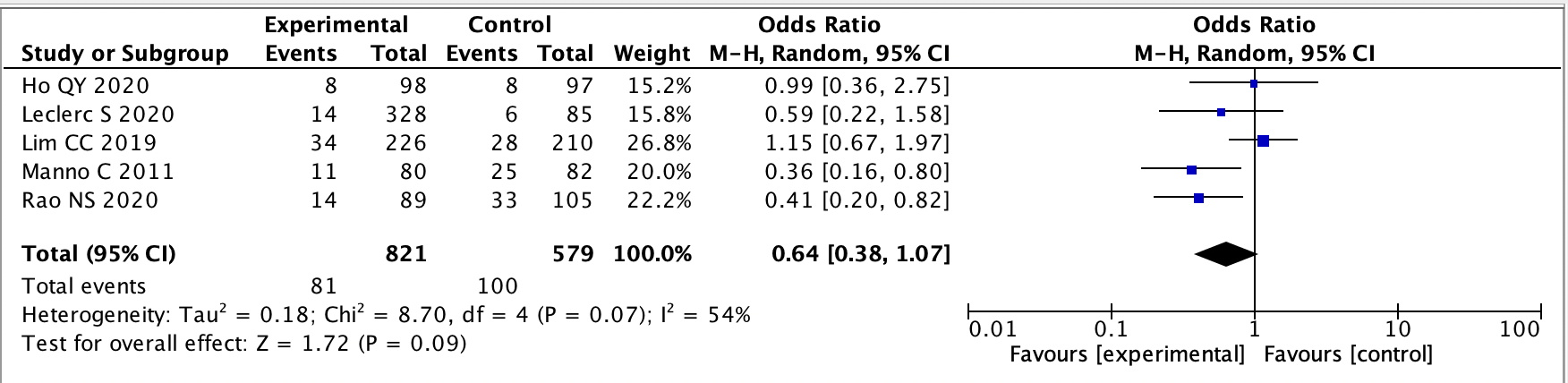

Supplement: Supplementary file 1 [file Presentation1.zip › Image 9.PNG]

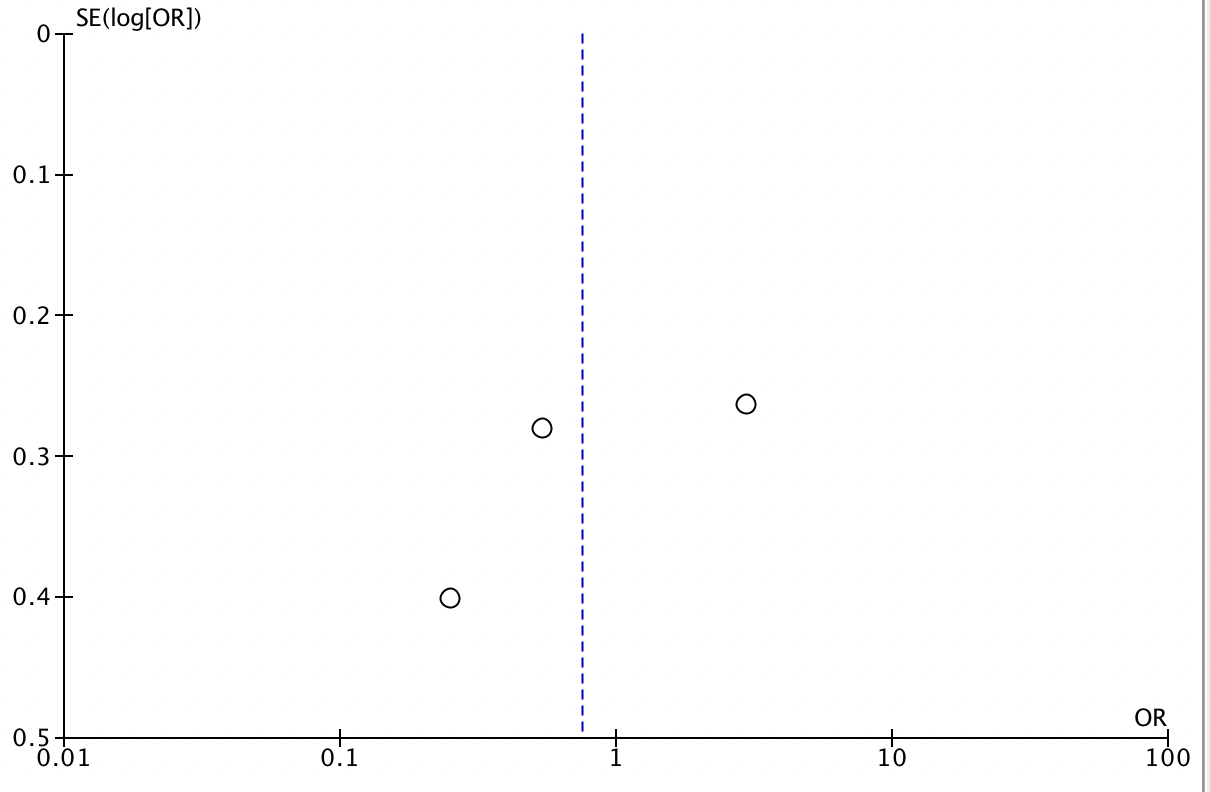

Supplement: Supplementary file 1 [file Presentation1.zip › Image 8.PNG]

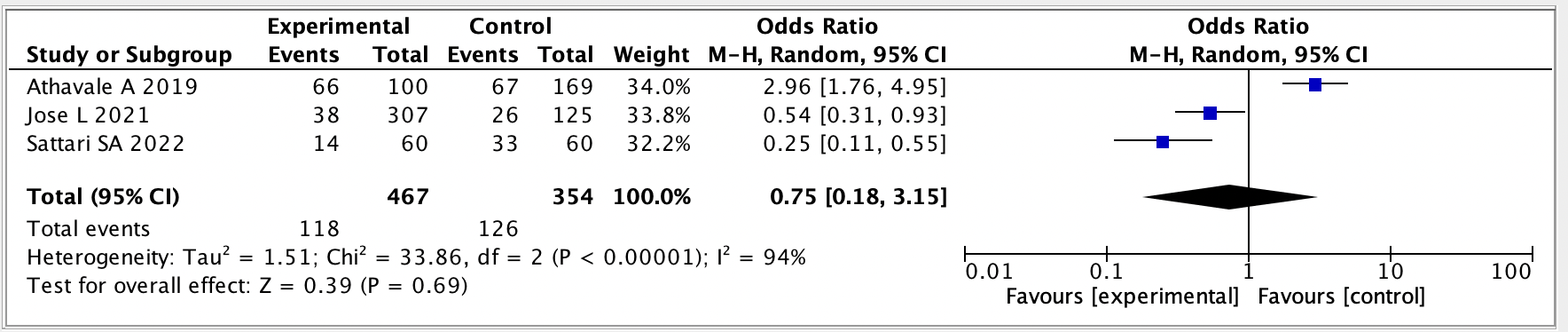

Supplement: Supplementary file 1 [file Presentation1.zip › Image 7.PNG]

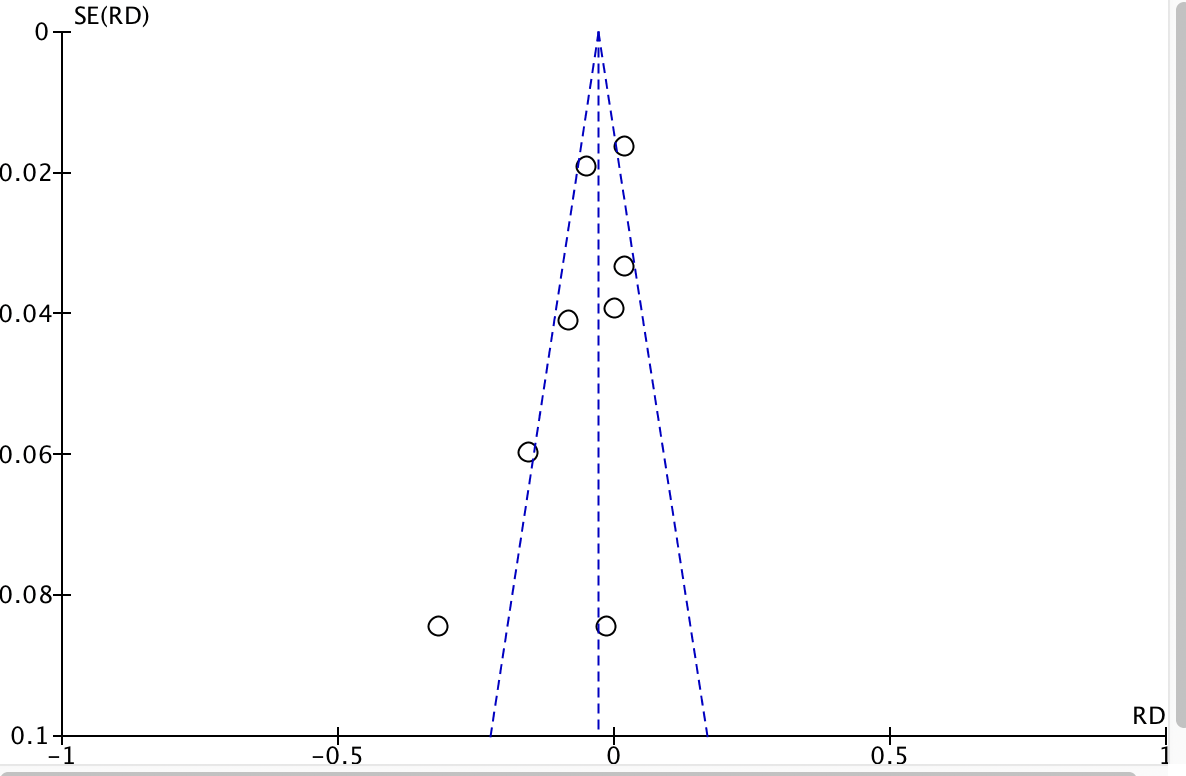

Supplement: Supplementary file 1 [file Presentation1.zip › Image 6.PNG]

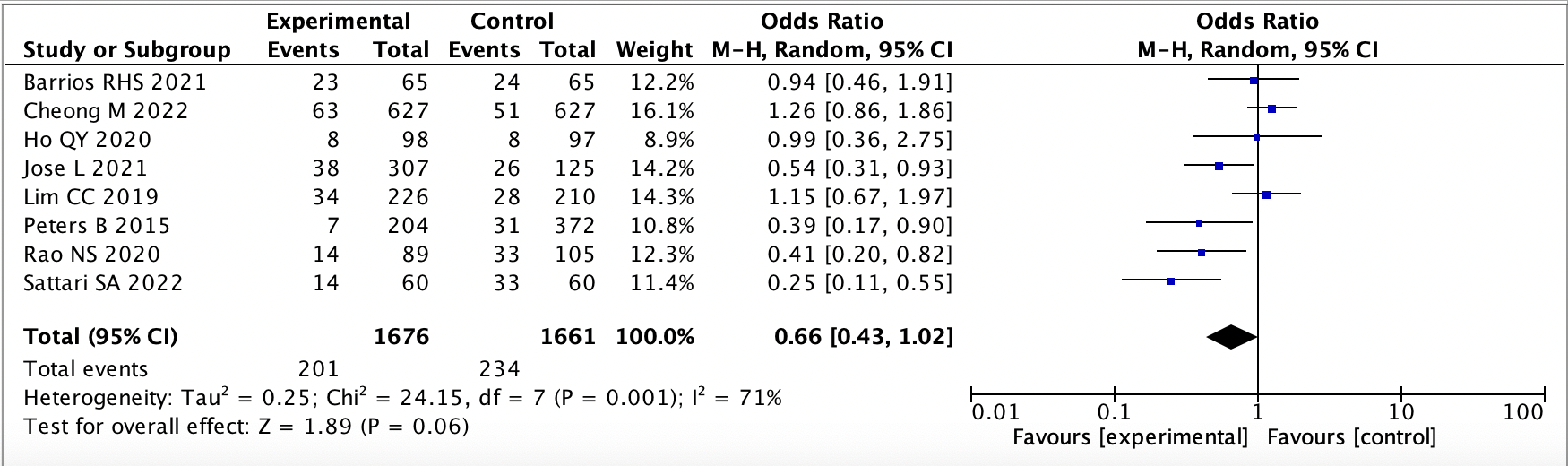

Supplement: Supplementary file 1 [file Presentation1.zip › Image 5.png]

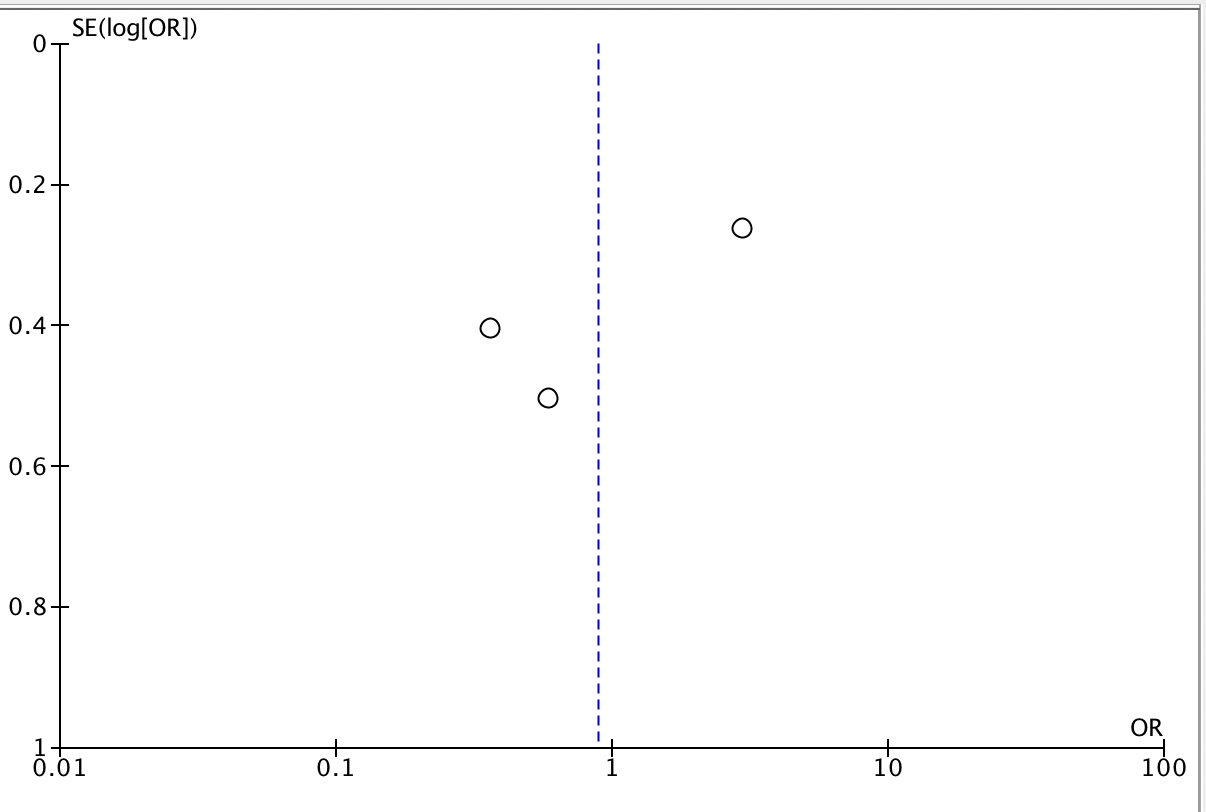

Supplement: Supplementary file 1 [file Presentation1.zip › Image 4.png]

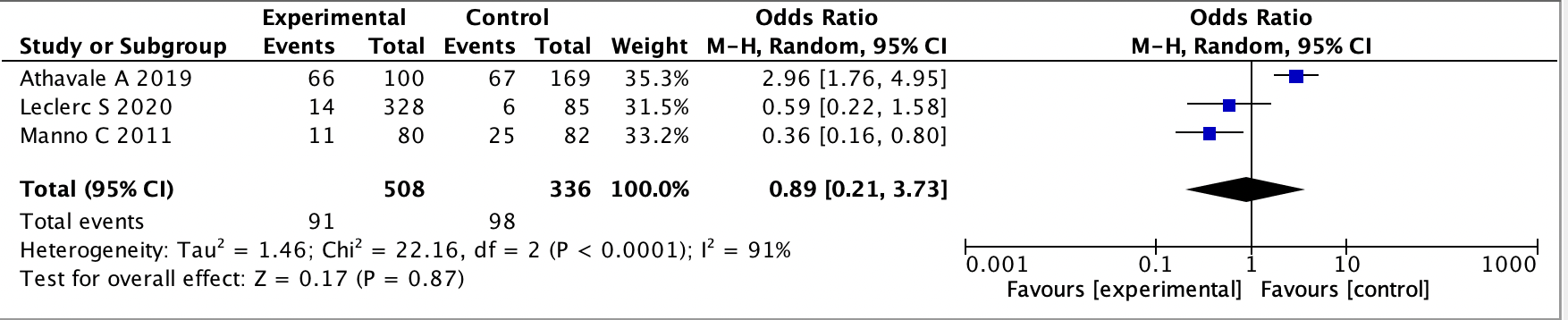

Supplement: Supplementary file 1 [file Presentation1.zip › Image 3.png]

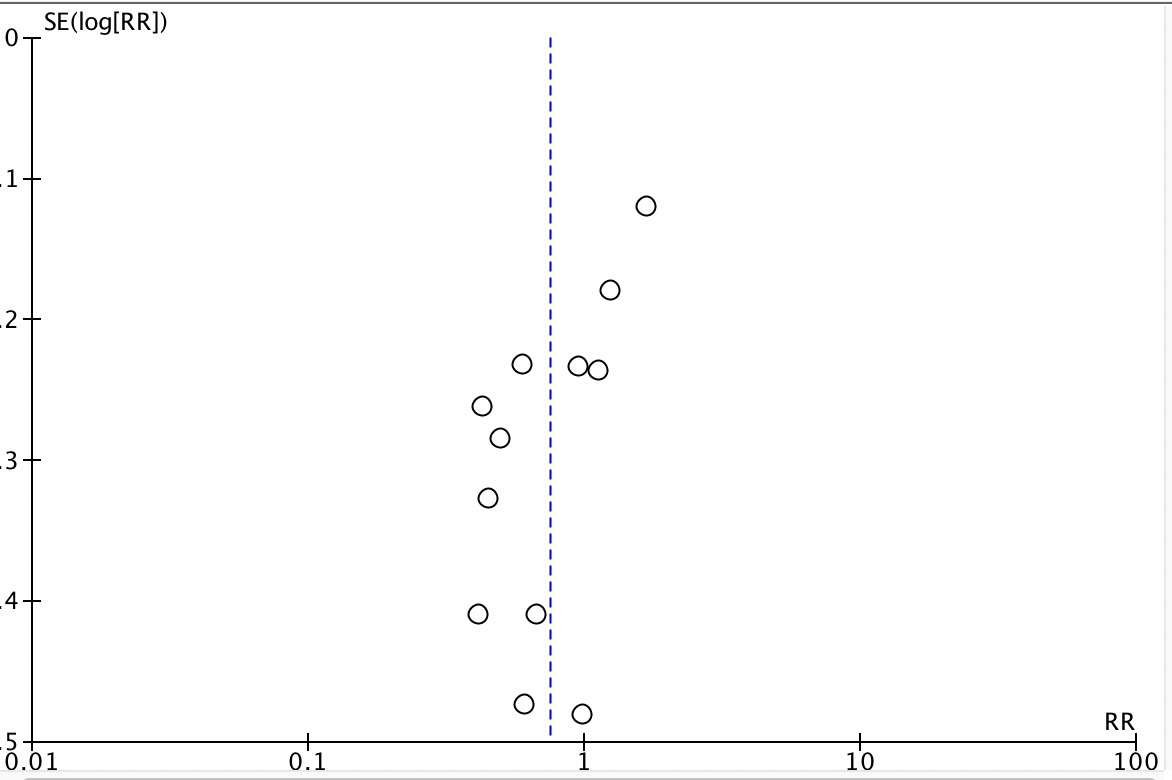

Supplement: Supplementary file 1 [file Presentation1.zip › Image 1.png]

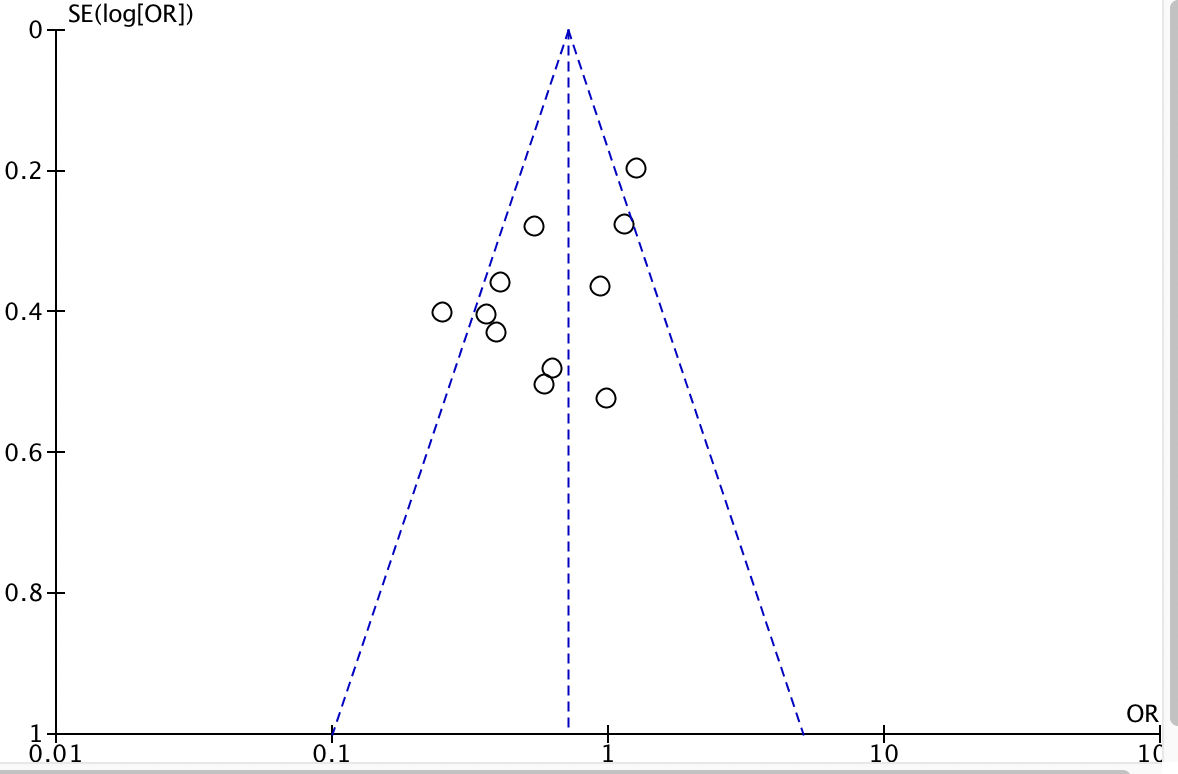

Supplement: Supplementary file 1 [file Presentation1.zip › Image 2.png]

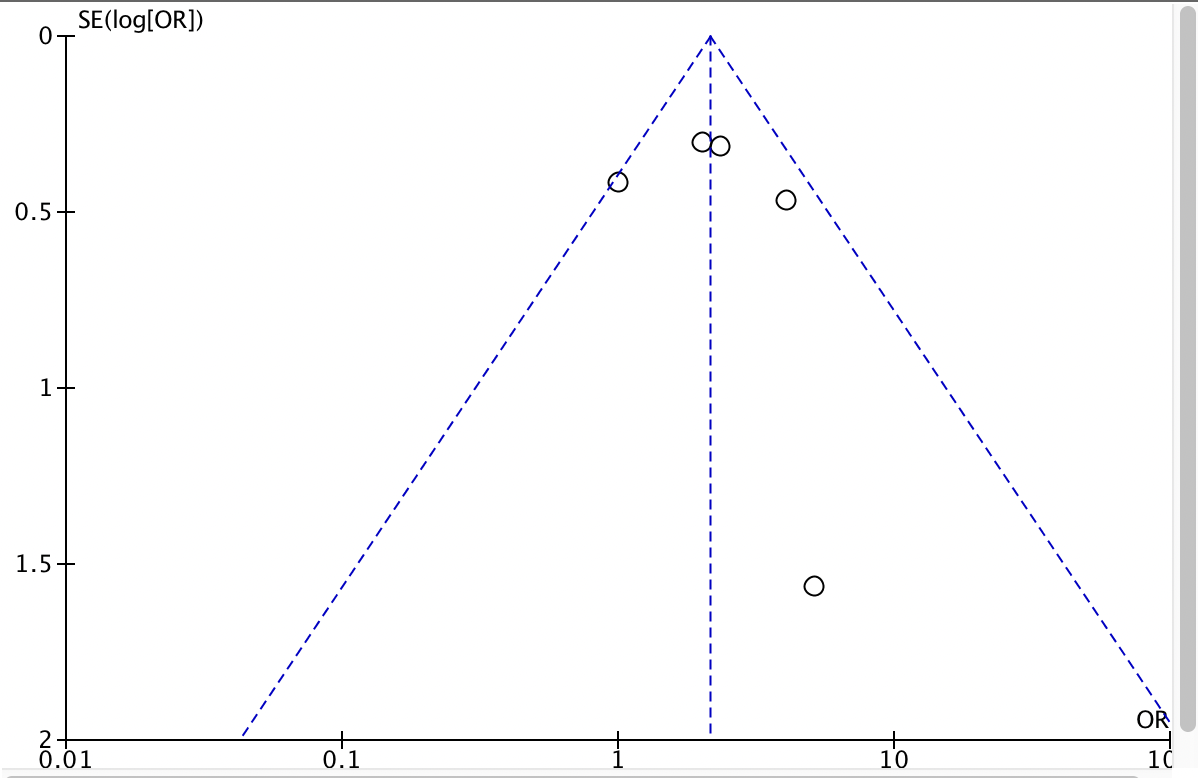

Supplement: Supplementary file 1 [file Presentation1.zip › Image 20.png]

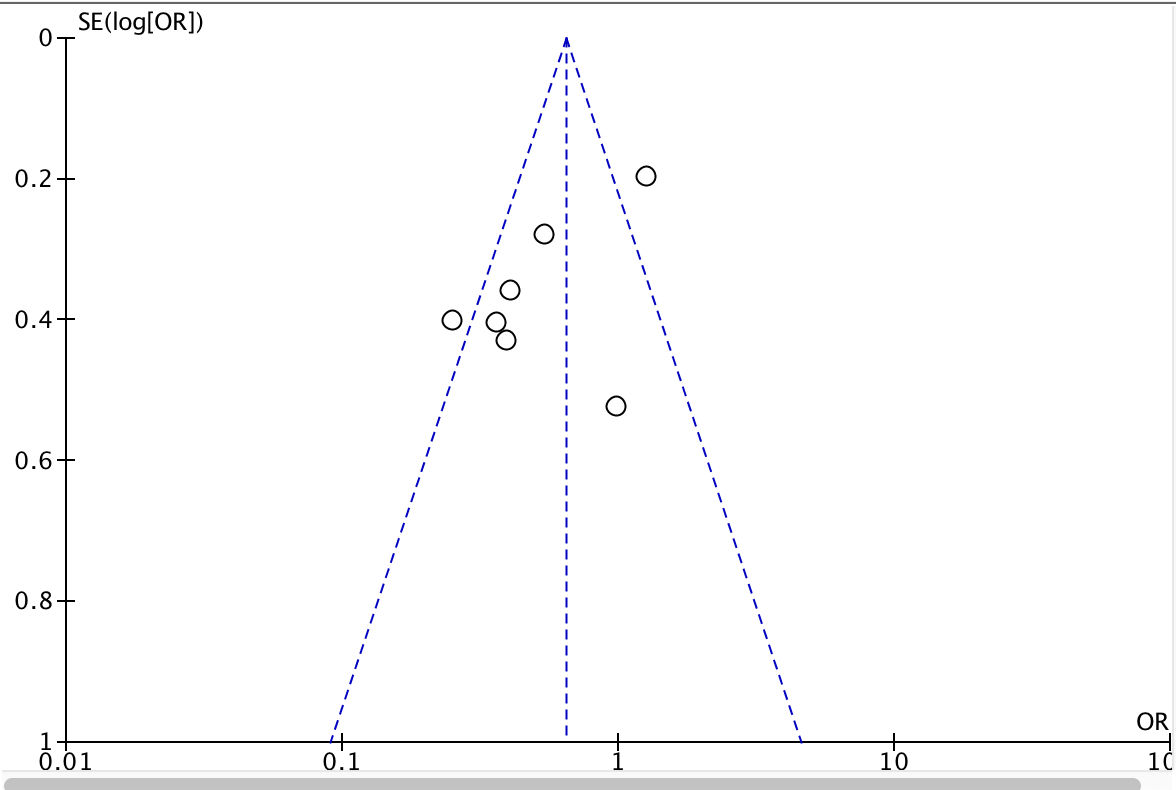

Supplement: Supplementary file 1 [file Presentation1.zip › Image 19.png]

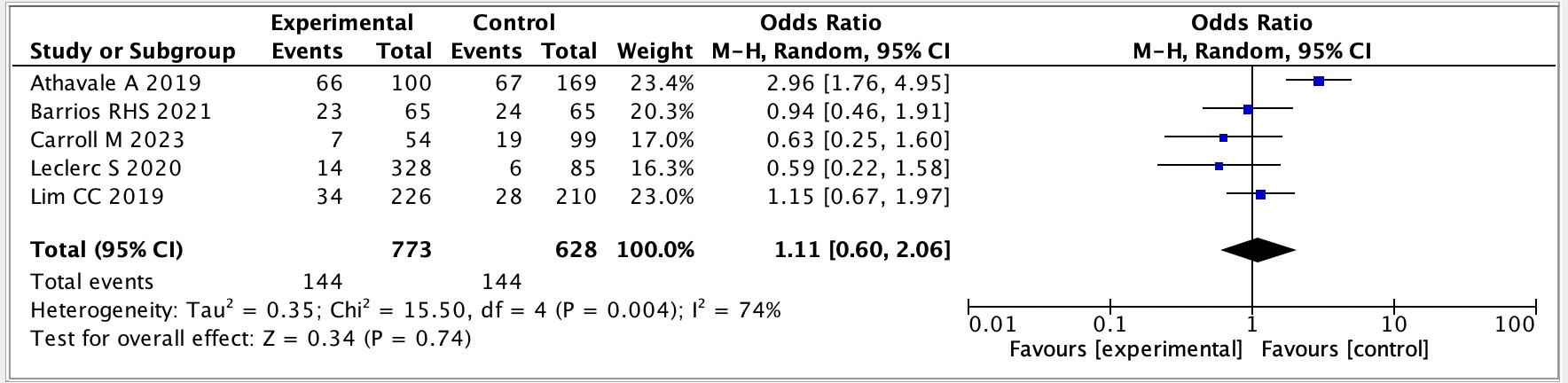

Supplement: Supplementary file 1 [file Presentation1.zip › Image 17.png]

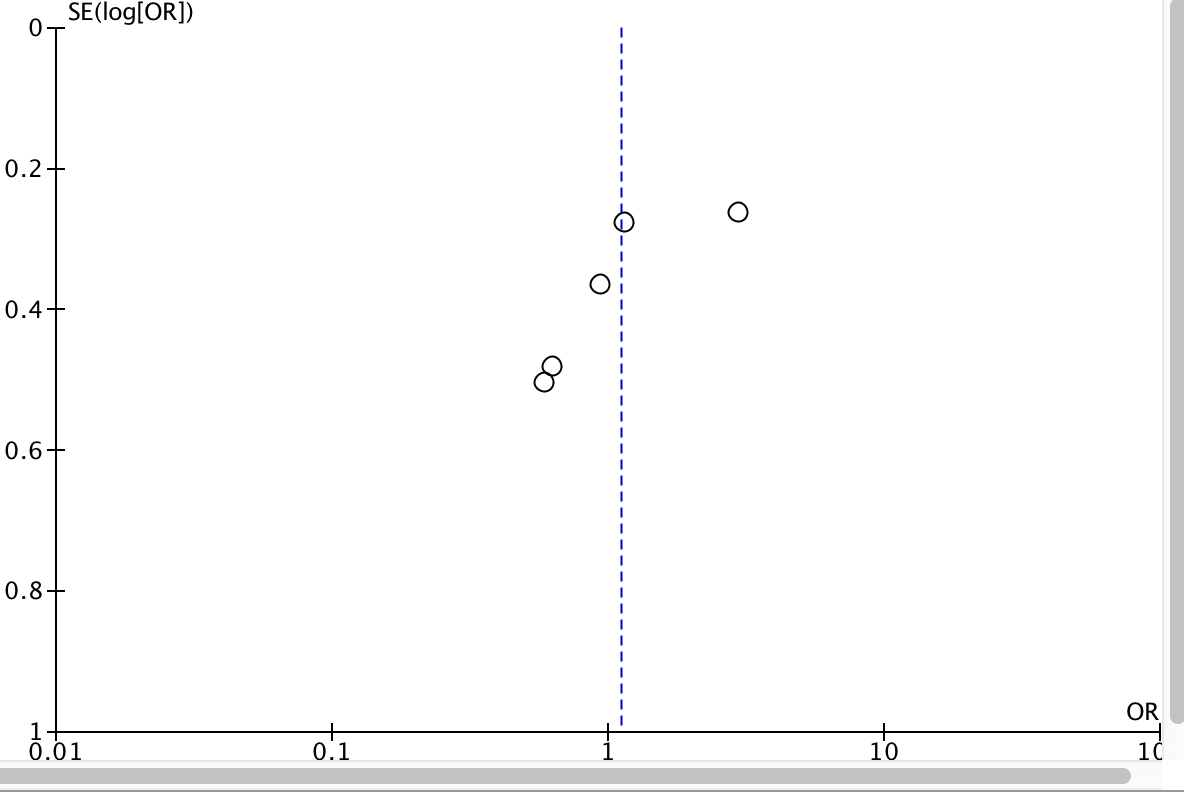

Supplement: Supplementary file 1 [file Presentation1.zip › Image 18.png]

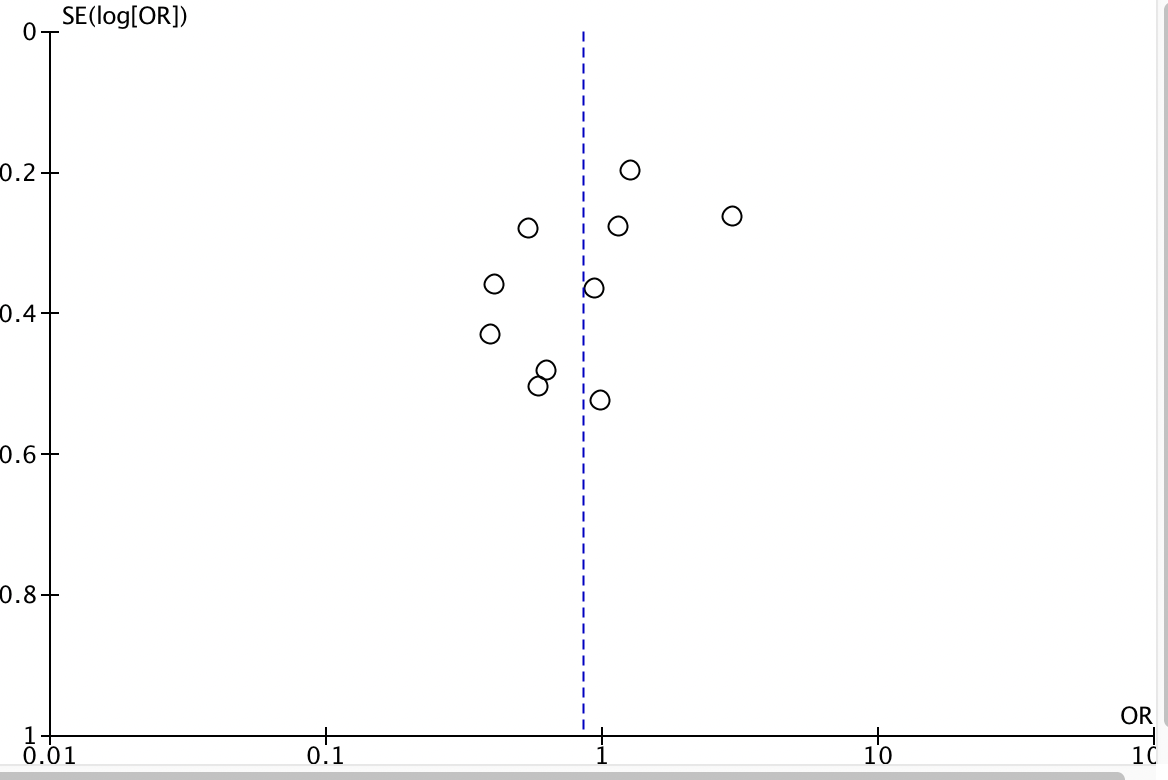

Supplement: Supplementary file 1 [file Presentation1.zip › Image 16.png]
