# Supplementary material for: Route-specific effects of desmopressin on bleeding and hyponatremia after kidney biopsy: meta-analysis of intranasal vs. intravenous administration
Source: Front Nephrol. 2025 Sep 23;5:1645418. doi: 10.3389/fneph.2025.1645418 (PMC12500430; doi:10.3389/fneph.2025.1645418)
Supplement: Supplementary file 2 [file Table1.docx]

**Supplementary Appendix**:

**PubMed Search strategy**:

(Deamino Arginine Vasopressin) OR (1-Desamino-8-arginine Vasopressin)) OR (Vasopressin, 1-Desamino-8-arginine)) OR (Desmopressin)) OR (Vasopressin, Deamino Arginine)) OR (1-Deamino-8-D-arginine Vasopressin)) OR (Vasopressin, 1-Deamino-8-D-arginine)) OR (Adiuretin SD)) OR (Apo-Desmopressin)) OR (DDAVP)) OR (Octostim)) OR (Desmotabs)) OR (Octim)) OR (Desmopressine Ferring)) OR (Ferring, Desmopressine)) OR (Desmospray)) OR (Nocutil)) OR (Desmopressin Acetate)) OR (Acetate, Desmopressin)) OR (Desmopressin Monoacetate, Trihydrate)) OR (Monoacetate, Trihydrate Desmopressin)) OR (Trihydrate Desmopressin Monoacetate)) OR (Desmopressin Monoacetate)) OR (Monoacetate, Desmopressin)) OR (Minirin)) OR (Minurin)) OR (Adiuretin)) OR (Desmogalen) AND (Kidney biopsy)) OR (Renal biopsy)) OR (biopsy of kidney)) OR (Renopuncture)) OR (renal puncture)) OR (Kidney puncture)) OR (Renipuncture)) OR (renal needle biopsy)

**Embase**

Mesh Desmopressin Free-text terms‘Deamino Arginine Vasopressin’ OR ‘1-Desamino-8-arginine Vasopressin’OR ‘Vasopressin, 1-Desamino-8-arginine’ OR ‘Desmopressin’OR ‘Vasopressin, Deamino Arginine’OR ‘1-Deamino-8-D-arginine Vasopressin’OR ‘Vasopressin, 1-Deamino-8-D-arginine’OR ‘Adiuretin SD’ OR ‘Apo-Desmopressin’ OR ‘DDAVP’ OR ‘Octostim’ OR ‘Desmotabs’OR ‘Octim’OR ‘Desmopressine Ferring’OR ‘Ferring, Desmopressine’ OR ‘Desmospray’ OR ‘Nocutil’OR ‘Desmopressin Acetate’ OR ‘Acetate, Desmopressin’OR ‘Desmopressin Monoacetate, Trihydrate’ OR ‘Monoacetate, Trihydrate Desmopressin’ OR ‘Trihydrate Desmopressin Monoacetate’OR ‘Desmopressin Monoacetate’OR ‘Monoacetate, Desmopressin’OR ‘Minirin’ OR ‘Minurin’OR ‘Adiuretin’OR ‘Desmogalen’

AND

Mesh ‘Kidney biopsy’Free-text terms‘Renal biopsy’OR ‘biopsy of kidney’OR ‘Renopuncture’OR ‘renal puncture’ OR ‘Kidney puncture’ OR ‘Renipuncture’OR ‘renal needle biopsy’
